# Dose-Dependent Effects of Cold Atmospheric Argon Plasma on the Mesenchymal Stem and Osteosarcoma Cells In Vitro

**DOI:** 10.3390/ijms22136797

**Published:** 2021-06-24

**Authors:** Artem M. Ermakov, Olga N. Ermakova, Vera A. Afanasyeva, Anton L. Popov

**Affiliations:** 1 Institute of Theoretical and Experimental Biophysics, Russian Academy of Sciences, 142290 Pushchino, Russia; beoluchi@yandex.ru (O.N.E.); va_vera_afanaseva@mail.ru (V.A.A.); antonpopovleonid@gmail.com (A.L.P.); 2 Kurnakov Institute of General and Inorganic Chemistry of the Russian Academy of Sciences, 119991 Moscow, Russia

**Keywords:** cold atmospheric argon plasma, low-dose, normal and cancer cells, cells proliferation, necrosis, apoptosis, gene expression, oxidative stress

## Abstract

The antimicrobial, anti-inflammatory and tissue-stimulating effects of cold argon atmospheric plasma (CAAP) accelerate its use in various fields of medicine. Here, we investigated the effects of CAAP at different radiation doses on mesenchymal stem cells (MSCs) and human osteosarcoma (MNNG/HOS) cells. We observed an increase in the growth rate of MSCs at sufficiently low irradiation doses (10–15 min) of CAAP, while the growth of MNNG/HOS cells was slowed down to 41% at the same irradiation doses. Using flow cytometry, we found that these effects are associated with cell cycle arrest and extended death of cancer cells by necrosis. Reactive oxygen species (ROS) formation was detected in both types of cells after 15 min of CAAP treatment. Evaluation of the genes’ transcriptional activity showed that exposure to low doses of CAAP activates the expression of genes responsible for proliferation, DNA replication, and transition between phases of the cell cycle in MSCs. There was a decrease in the transcriptional activity of most of the studied genes in MNNG/HOS osteosarcoma cancer cells. However, increased transcription of osteogenic differentiation genes was observed in normal and cancer cells. The selective effects of low and high doses of CAAP treatment on cancer and normal cells that we found can be considered in terms of hormesis. The low dose of cold argon plasma irradiation stimulated the vital processes in stem cells due to the slight generation of reactive oxygen species. In cancer cells, the same doses evidently lead to the formation of oxidative stress, which was accompanied by a proliferation inhibition and cell death. The differences in the cancer and normal cells’ responses are probably due to different sensitivity to exogenous oxidative stress. Such a selective effect of CAAP action can be used in the combined therapy of oncological diseases such as skin neoplasms, or for the removal of remaining cancer cells after surgical removal of a tumor.

## 1. Introduction

Cold atmospheric plasma (CAP) has found its application in medicine as an effective agent for microflora destruction on various surfaces or in liquids [1]. This agent can directly affect mammalian cells and tissues and provide a significant reduction in microbial load (for example, on wound surfaces), including multiple antibiotic-resistant bacteria [2]. CAP is effective not only for a microorganism’s destruction, but also for viruses, protozoa and even the destruction of parasitic worms [3,4,5].

Currently, cold plasma technology is used in dermatology, dentistry, oncology and skin infection treatment [6]. The most promising application area of plasma therapy is the treatment of non-healing wounds and ulcers [7]. The CAP use in oncology also shows positive results in preclinical and clinical trials. In contrast to chemotherapy and radiation therapy, the use of cold plasma for medical purposes is not accompanied by side effects, which is confirmed by preclinical and clinical data [8,9,10]. Large amounts of reactive oxygen and nitrogen species (ROS/RNS) such as ozone, atomic oxygen, superoxide anion, hydroxyl radical, hydrogen peroxide, nitric oxide, and peroxynitrite are formed in a plasma stream [11]. ROS and RNS are formed by the interaction of active plasma particles with air and/or liquid. Herewith, hydroxyl radicals and nitrogen oxides are formed in the air phase, while nitrites and nitrates are formed as a result of the interaction of plasma with liquid. It is believed that ROS, RNS and hydrogen peroxide are among the major factors affecting cells [12]. Furthermore, the UV, VIS, and IR (heat), as well as electromagnetic fields and free electrons also play a certain role in the biological efficiency of cold plasma [13].

It has been shown that low doses of CAP irradiation activate the processes of cell proliferation and wound healing [14]. High CAP irradiation doses activate an oxidative stress in eukaryotic cells, causing apoptosis and necrosis [15]. Meanwhile, different types of cells (normal or cancer) should specifically react to this factor. It has been shown that cancer cells are more sensitive to low intensity oxidative stress than normal cells [16]. Currently, there are several types of cold plasma generators in biomedical research, including those based on direct-discharge, based on indirect-discharge, and hybrid types [17]. Plasma exposure can be direct, under in vitro conditions or in experiments on animals or human tissues where plasma irradiation directly interacts with the object. In the case of a solution or liquid medium treatment with cold plasma, an indirect exposure takes place. An activated liquid medium can be used for cell cultivation or for injection directly to animals [18]. Thus, depending on the generator type and experimental conditions, the active factors of CAP treatment may vary significantly. That is why it is difficult to adequately compare the CAP effects on different cell types. At the same time, there are virtually no studies showing the efficiency of cold plasma exposure under the same irradiation conditions, using the same generators, but on different cell types.

In this work, we investigated the sensitivity of normal and cancer human cells to low and high irradiation doses of cold atmospheric argon plasma (CAAP) and under different irradiation modes. We have shown that CAAP stimulates the processes of proliferation in stem cells at the same small doses of irradiation, whereas in cancer cells, it leads to a significant slowdown in proliferation, apoptosis activation, and cell necrosis. The high-dose CAAP treatment is detrimental to both stem and cancer cells. The obtained results can be used in the development of methods and modes of CAAP application for the selective destruction of cancer cells, for example, skin neoplasms or for the removal of remaining cancer cells after surgical removal of a tumor. The CAAP use would allow this, not only without damaging normal cells, but also with the stimulation of the processes of their proliferation and growth, thus providing recovery of damaged human tissues after surgical procedures.

## 2. Results

### 2.1. The Effect of CAAP on the Proliferation Rate and an Apoptosis of Normal and Cancer Cell Lines

**Figure 1:**
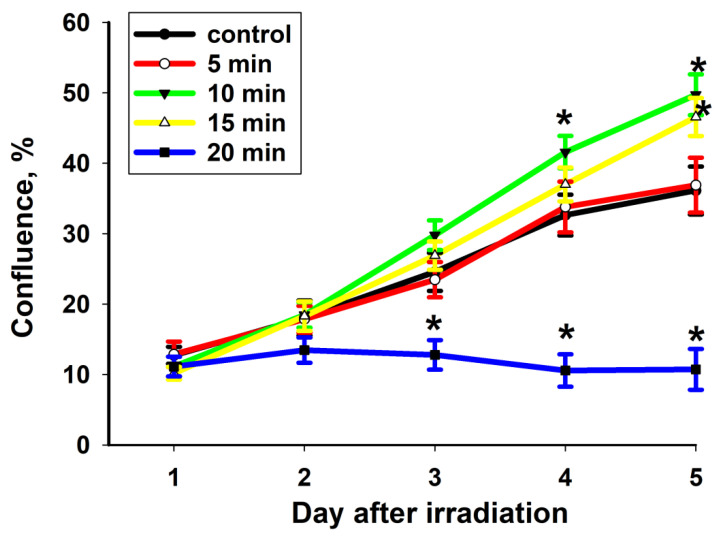
The density-increase dynamics of normal MSC cells after CAAP treatment at different exposure times (5 min, 10 min, 15 min and 20 min) and in the control experiment. * *p* < 0.001.

The proliferation rates of MSCs after CAAP irradiation are shown in Figure 1. CAAP irradiation of MSCs for 5 min did not lead to any changes in proliferation activity. The exposure duration when increased to 10 min enhanced the proliferation rate on the fourth and fifth days after CAAP irradiation (by 28% and 37%, respectively). The average confluence value was 29% higher than the control value after 15 min of CAAP irradiation on the fifth day of cultivation. A further increase in the irradiation duration to 20 min led to a significant inhibition of cell culture growth (by 47–70%) starting from the third cultivation day.

**Figure 2:**
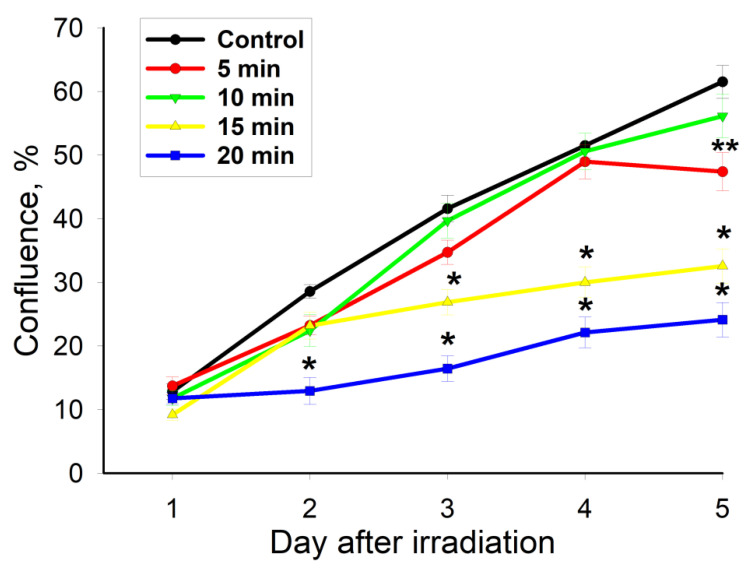
The density-increase dynamics of normal MSC cells after CAAP treatment at different exposure times (5 min, 10 min, 15 min and 20 min) and in the control experiment. * *p* < 0.001, ** *p* < 0.01.

The CAAP exposure of MNNG/HOS osteosarcoma cells for 5 min did not affect their proliferation dynamics at the beginning of cultivation. However, the proliferation of MNNG/HOS cells is inhibited as confirmed by a 23% slowdown on the fifth day after exposure (Figure 2). The proliferation of cancer MNNG/HOS cells was not accelerated after 10 min of CAAP irradiation in contrast to MSCs, but the tendency to inhibition was less pronounced.

A further increase in the CAAP exposure time to 15 min led to a significant inhibition of MNNG/HOS proliferation on the third, fourth, and fifth days after irradiation (by 25%, 32% and 41%, respectively). Exposure to CAAP for 20 min resulted in a significant decrease (by 60–67%) in the proliferation rate of MNNG/HOS cells starting from the second day after treatment. One of the hypotheses of such a pronounced selectivity of CAAP irradiation against MNNG/HOS osteosarcoma cells may be the decreased activity of the antioxidant system. It is well known that, compared to normal cells, cancer cells are more vulnerable to ROS accumulation due to their increased production rate, where they contribute to several aspects of their proliferation and metastasis [19]. It has been previously shown that antioxidant supplementation (N-acetyl cysteine) increases proliferation of CAAP-treated osteosarcoma cells, implicating an involvement of redox signaling and expression of PRX1/PRX2 [20]. Thus, it can be concluded that the regulation of the cell redox status is one of the possible molecular mechanisms for the regulation of apoptosis induced by CAAP irradiation.

### 2.2. The Effect of CAAP on a Cell Circle, Apoptosis and Necrosis of Normal and Cancer Cells

**Figure 3:**
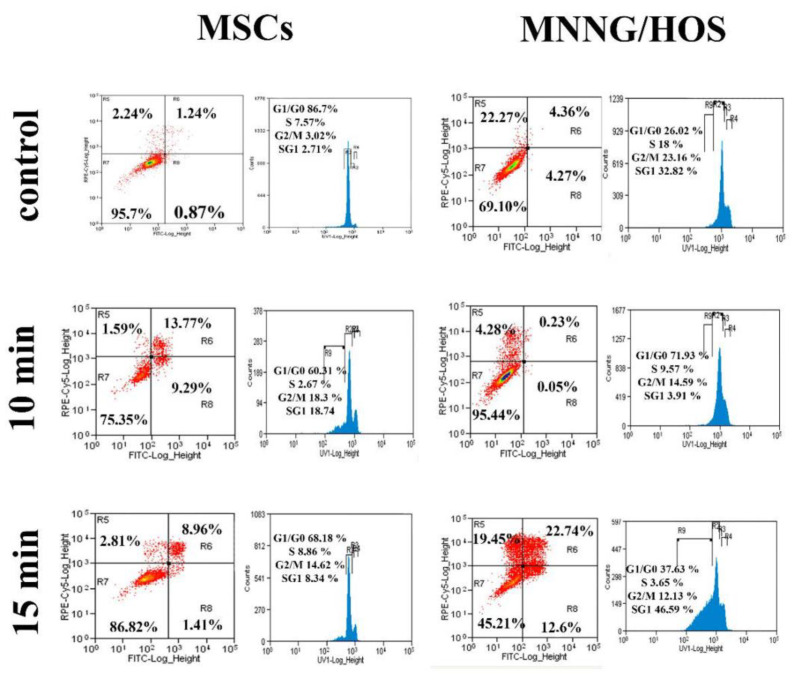
The flow cytometry analysis of cells (MSCs and MNNG/HOS) 72 h after CAAP irradiation with 10 and 15 min exposure time. The red dots on the left side of each diagram show the distribution of cells by quadrant depending on R5—necrosis, R6—late apoptosis, R7—living cells, R8—early apoptosis. The histograms show cell distribution by cell cycle phases: G_1_/G_0_, S, G_2_/M, and SG_1_ (G_1_ subphase). The digital data on the histograms are the average values for three independent experiments.

Figure 3 shows that CAAP irradiation of stem cells leads to their partial death. The rate of the cell death depends on the exposure time and the cultivation duration after irradiation. Cell death is caused mainly by apoptosis. In the MSC population, the number of cells in early and late apoptosis increased slightly on the first and second days of cultivation after 10 min of CAAP treatment (Figure S1). On the third day (72 h), it increased on average to 10% (*p* < 0.01). In contrast, the number of cells in necrosis decreased.

A longer 15 min CAAP irradiation of MSCs contributed mostly to an increase in the number of cells in late apoptosis and it had less impact on the cells in early apoptosis. The number of necrotic cells did not change. The maximum number of cells in late apoptosis was observed on the first day after irradiation (*p* < 0.01). Then, their number decreased and increased again by the third cultivation day.

Changes in the MSCs cell cycle initiated by 10 min and 15 min CAAP treatment resulted in a significant increase, by three to five times on average (*p* < 0.001), in the number of G2/M phases cells. In the latter case, a fraction of sub-G1 phase cells appeared as a consequence of greater cell death.

Figure S1, b shows that CAAP treatment of MNNG/HOS cell culture results in their massive death. Exposure to CAAP for 10 min was marked by cell death mainly caused by necrosis, unlike stem cells. In the case of 15 min exposure time, the intensity of cancer cell death increased sharply. An avalanche-like growth of cells in early and late apoptosis and necrosis is observed. As a result, on the third day the number of dying and dead cells reached 55% (*p* < 0.001) of the total cells in the analyzed populations.

After 10 min CAAP irradiation of cancer cells, the number of cells in G2/M phases increased significantly (*p* < 0.001). However, there was a general decrease in cell proliferative activity: the S phase cell fraction decreased by two times (*p* < 0.001) and the G2/M phase cell fraction decreased by 1.4 times (*p* < 0.05) on the third day after irradiation. An increase in the CAAP irradiation duration of MNNG/HOS cells to 15 min was accompanied by even more significant changes in the cell cycle. After a day, the proportion of S phase cells increased by 1.6 times (*p* < 0.01), but the number of G2/M phase cells decreased by 2.3 times (*p* < 0.001). Due to the mass death of MNNG/HOS cells, the proportion of sub-G1 phase cells increased noticeably (by 2.5 times). Two days after irradiation, the sub-G1 phase cell fraction increased significantly (by 6.5 times, *p* < 0.001), while G2/M phase cell fraction increased by 2.2 times, *p* < 0.01. On the third day after irradiation, the general decrease in proliferative activity was expressed in the reduction of S phase cell proportion (by 5.3 times, *p* < 0.001), and G2/M phases cell proportion (by 1.7 times, *p* < 0.001). At the same time, the number of necrotic cells and, accordingly, the number of sub-G1 phase cells increased (by 2.3 times, *p* < 0.001).

**Figure 4:**
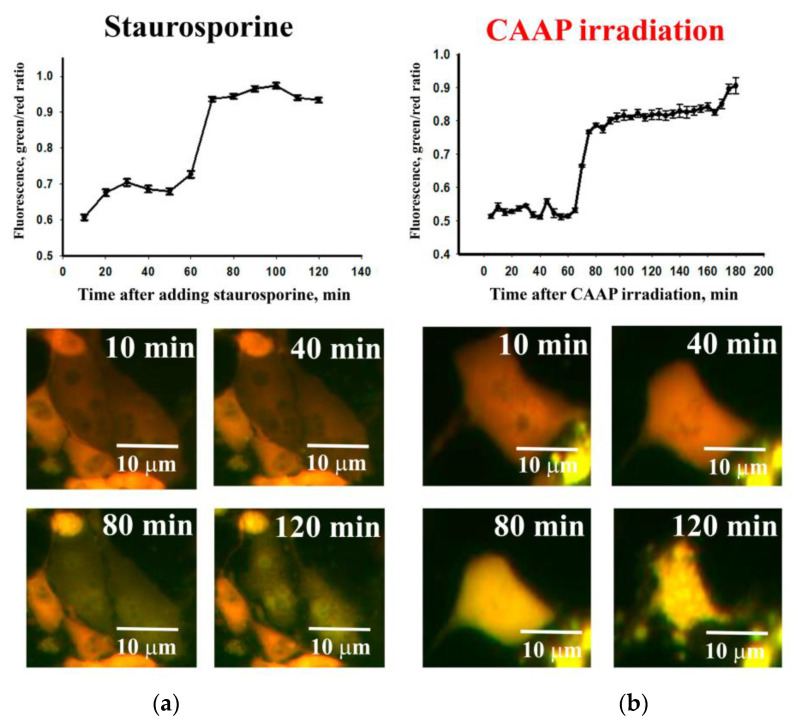
The analysis of MNNG/HOS cell death. Apoptosis was detected by a change in the ratio of fluorescence intensity in the green and red spectrum of the Casper3-GR protein—a sensor of caspase-3-dependent apoptosis: the dynamics of cancer cells death after staurosprine treatment (**a**); the dynamics of cancer cells death after 15 min exposure to CAAP (**b**).

As FACS plots analysis showed, cancer cell death occurs either by necrosis or apoptosis. The mechanism of apoptosis death of MNNG/HOS cells was investigated using intracellular sensor of caspase-3-depending apoptosis—fluorescing protein Casper3-GR. As a positive control, we used staurosporine for apoptosis activation (Figure 4a). The injection of this substance to the medium with MNNG/HOS cell culture led to a caspase-3-depending apoptosis activation after 60 min of incubation (Figure 4b). It was expressed by a sharp increase in green cytoplasm fluorescence. After 15 min of CAAP irradiation, there was a sharp increase in green fluorescence (activation of caspase 3) in cancer cells after a 60 min incubation. In the next 30 min after caspase 3 activation, the cells were destroyed (Figure 4b).

Considering that CAAP exposure leads to the formation of ROS and RNS not only in the cell cytoplasm, but outside the cell membrane, we can propose that the effect of CAAP-irradiation substances, such as hydrogen peroxide or peroxynitrite, applies to most important membrane channels, pores and integral proteins. Thus, one of the possible mechanisms of CAAP anticancer effects may be the effect of CAAP-irradiation substances on the degradation processes of integral membrane proteins involved in anti-apoptosis and proliferation signaling. Earlier, it has been shown that the CAAP-treatment of oral squamous cell carcinoma, which is an overexpression of epidermal growth factor receptor (EGFR), led to intracellular ROS increase and a decrease of non-enzymatic antioxidants which led to the degradation and dephosphorylation of the EGFR via formation of oxidative thiol residues [21]. The loss of such an important integral protein leads to the death of cancer cells through the apoptosis mechanism. At the same time, such a CAAP treatment of normal human gingival fibroblasts did not cause their death and the inhibition of proliferation.

### 2.3. The Effect of CAAP on a Cell Circle, Apoptosis and Necrosis of Normal and Cancer Cells

**Figure 5:**
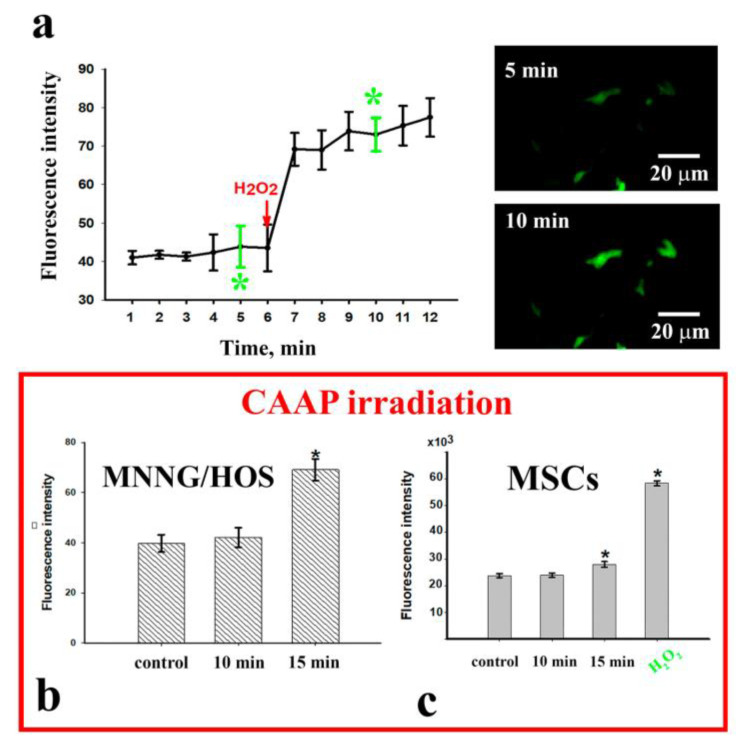
ROS formation in transfected MNNG/HOS cells detected by the level of HyPer sensor protein fluorescence in the cytoplasm and the level of the H_2_DCFDA dye fluorescence in MSCs: (**a**) the dynamics of the changes in the cell fluorescence before and after the addition of 100 µM of hydrogen peroxide (positive control); (**b**) change in the fluorescence of MNNG/HOS cells after CAAP irradiation; (**c**) change in the fluorescence of MSCs after CAAP irradiation. * p < 0.001.

We observed an increase in the HyPer protein fluorescence intensity by 1.67 times (*p* < 0.001) (Figure 5a) 6 min after the introduction of 100 µM of hydrogen peroxide into the medium (positive control). The increased level of fluorescence was maintained for at least 6 min (Figure 5b). The CAAP treatment of MMNG/HOS cells for 10 min did not lead to an increase in the intensity of HyPer protein green fluorescence in the cell cytoplasm (Figure 5b). The longer exposure time of 15 min (resulting in the death of more than half of the cells) was accompanied by a significant increase in the intensity of cell fluorescence by 1.7 times (*p* < 0.001) compared to the control.

Similar results were obtained during the CAAP treatment of MSCs previously incubated with H_2_DCFDA (Figure 5c). The 10 min treatment of cells in a cold plasma stream did not lead to an increase in fluorescence, whereas 15 min irradiation was accompanied by a significant increase in the intensity of the fluorescent signal (by 23%) as a consequence of ROS generation in cells. When 100 µM of H_2_O_2_ were added to the medium, the cell fluorescence intensity increased by 2.5 times after 30 min, compared to the untreated control.

The different levels of intracellular ROS in normal and cancer cells after CAAP irradiation can be explained not only by the different redox status and the rate of cellular ROS metabolism, but also by the increased ability of cancer cells to diffuse the extracellular hydrogen peroxide generated by CAAP in a water microenvironment through the aquaporin system. Previously, using human glioblastoma cells, it has been shown that the accelerated diffusion of H_2_O_2_ through the cytoplasmic membrane via aquaporins one, three, and eight leads to a significant increase in the level of intracellular ROS, which ultimately leads to apoptosis [22]. At the same time, a similar duration of CAAP irradiation of normal astrocytes did not cause such an increase in intracellular ROS and did not cause their death via the apoptosis pathway. Thus, CAAP irradiation can provide a selective cytotoxic effect on cancer cells by different diffusion efficiency through the cytoplasmic membrane of water photolysis products.

### 2.4. The Effect of CAAP on Genes Expression in Normal and Cancer Cells

**Figure 6:**
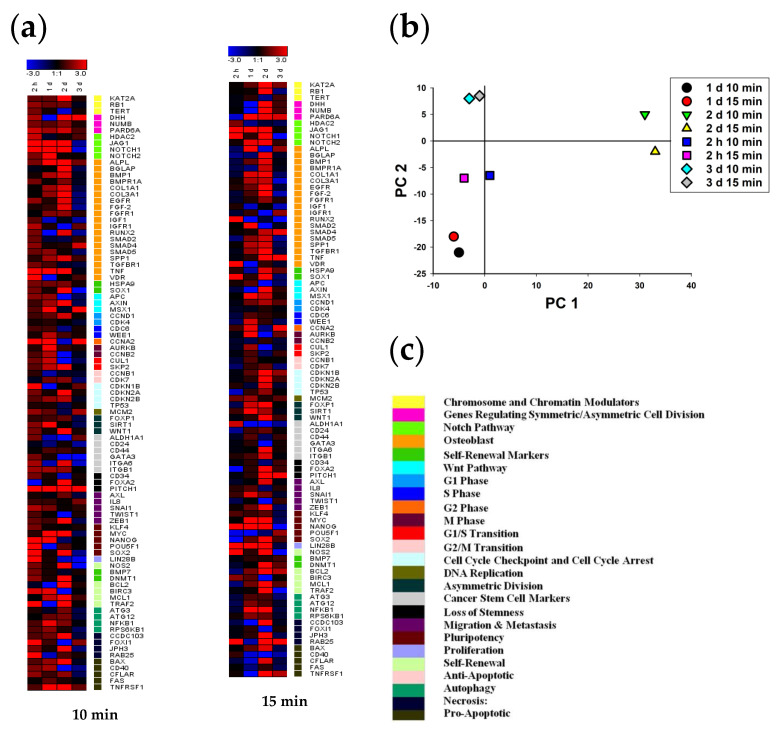
Heat map of gene expression in MSCs treated with 10 and 15 min CAAP irradiation after 2 h, 24 h, 48 h and 72 h of incubation (the deviations are shown in a logarithmic scale with a base of 2): (**a**) the intensity scale of the standardized expression values ranges from −3 (green: low expression) to +3 (red: high expression, with 1:1 intensity value (black) representing the control (nontreated); (**b**) principal component analysis (PCA) of qRT-PCR data for MSCs treated with 10 and 15 min CAAP irradiation after 2 h, 24 h, 48 h and 72 h; (**c**) cluster groups of genes and their functionality. The belonging of genes to different clusters and markers is shown using the color scale which is to the right of the diagrams. A validated database for gene ontology annotations is presented in Table S1.

Figure 6a shows that the changes in the genes’ transcriptional activity have a similar pattern after both 10 and 15 min irradiation. Groups, depending on the observed period, form common clusters of gene expression. In particular, as shown in Figure 6a, an initial burst of transcription was observed 2 h after irradiation. A day after irradiation, the level of gene expression increased in clusters of chromatin and chromosome modulators, regulators of symmetric cell division, NOTCH signaling, WNT signaling, markers of cell proliferation, markers of autophagy and proapoptotic factors. At the same time, the expression level typically did not differ, or was lower than the control in the other groups of genes, remaining high only in a few genes. In particular, in the cluster of osteogenic differentiation markers, the ALPL and TNF gene expression remained high, while the expression level of the RUNX2 and VDR genes was significantly lower than the control. In the group of asymmetric division markers, the expression of the SIRT1 gene was additionally increased many times. On the contrary, in the group of markers of stem reduction, a high level of transcription was maintained only in the PITCH1 gene. Among the pluripotency markers, the expression of the MYC gene significantly increased and the transcription level of the NANOG gene remained high, while among the anti-apoptotic markers the activation of BIRC3 and MCL1 genes continued. PCA analysis is shown in the Figure 6b.

On the second day after MSC exposure to CAAP, a further increase in transcription activation was observed in the osteogenic differentiation cluster (for the ALPL, BGLAP, BMP1, BMPR1A, COL1A1, COL3A1, EGFR, FGF-2, FGFR1, RUNX2, SPP1, and TNF genes). The level of gene transcription in the clusters of chromatin and chromosome modulators and symmetric cell division regulators was increased too. A massive increase in the level of transcription continued in the majority of genes in the group of asymmetric division markers, falling stemness markers, antiapoptotic markers, and autophagy markers. In other groups, high transcription levels were maintained in single genes ‒ MYC and SOX2 (pluripotency markers), CCDC103 and JPH3 (necrosis markers), BAX, CFLAR, and TNFRSF1 (proapoptotic markers).

On the third day after cell exposure to CAAP, there was a decrease in transcriptional activity to the control level or below in almost all the studied gene groups. Residual high expression activity was observed in the cluster of symmetric division regulators DHH and PARD6A, in the cluster of osteogenic differentiation—ALPL, IGFR1 and SMAD4, in the WNT signaling group—MSX1, and among the markers of stem reduction—PITCH1. A decrease in transcript levels was observed for the JAG1 and NOTCH1 genes (NOTCH signaling), BGLAP, COL3A1, and EGFR. FGF-2, IGF1, RUNX2, and SMAD5 genes (osteogenic differentiation), and for single genes in other clusters (SOX1, APC, CDK7, SIRT1, CD24, GATA3, ITGA6, SOX2, LIN28B, NOS2, RPS6KB1, JPH3, CD40, and CFLAR).

Upon the increase in MSC exposure to CAAP to 15 min, the gene expression patterns changed differently. Thus, after 2 h, instead of generalized activation of expression, an increase in the transcription level was detected only in single genes in the groups of NOTCH signaling (HDAC2, JAG1, and NOTCH1), osteogenic differentiation (RUNX2 and VDR), self-renewal markers (SOX1), pluripotency (NANOG), proliferation, antiapoptotic markers, and necrosis (LIN28B, NOS2, BIRC3, and RAB25).

On the first day after 15 min exposure to cold plasma, there was an increase in the expression level of a significant number of genes in the osteogenic differentiation cluster (ALPL, BMP1, COL1A1, COL3A1, EGFR, SMAD5, SPP1, and TGFBR1) and cell proliferation markers (CCND1, CDC6, WEE1, CCNA2, AURKB, CUL1, SKP2, CCNB1, CDK7, and MCM2). The transcription level of asymmetric division, antiapoptosis and autophagy markers was also increased in most genes. In contrast, the expression of most genes was significantly lower than the control level in the group of proapoptotic markers. In other groups, the amount of mRNA exceeded the control level in only a few genes (KAT2A, PARD6A, JAG1, NOTCH2, MSX1, IL8, and SNAI1). For the DHH, NUMB, AXIN, ALDH1A1, CCDC103, and RAB25 genes it did not differ from the control level, or even decreased.

In the following two days, there was a significant increase in the level of transcription of most genes in clusters of chromatin and chromosome modulators, regulators of symmetric cell division, NOTCH signaling, osteogenic differentiation, self-renewal, WNT signaling, checkpoint markers, asymmetric division, decreased stemness, pluripotency, antiapoptotic markers, autophagy, and necrosis markers. At the same time, the expression level was increased only in the CCND1, CDK7, and LIN28B genes in the proliferation marker group. The transcription level did not differ from the control or was reduced in the remaining genes.

On the third day after 15 min of CAAP exposure, the cells showed a drop in the expression level of most genes to the values that did not differ from the control ones. The increased transcription was observed in only 16 of the 96 genes: KAT2A, PARD6A, ALL, SMAD4, TNF, HSPA9, CCND1, CCNA2, AURKB, CDKN1B, MCM2, PITCH1, DNMT1, BCL2, RAB25, and TNFRSF1. The transcription level was significantly lower than the control one in five genes (NOTCH1, RUNX2, NANOG, NOS 2, and TRAF2).

Unlike the stem cells, the transcriptional gene activity in MNNG/HOS cells changed after CAAP irradiation in a different way. As Figure 6b shows, the gene expression was significantly different at the second hour after 10 min CAAP irradiation. Gene transcription patterns after 10 min of CAAP irradiation on first, second and third days were similar, whereas the 15 min exposure duration resulted in significant changes in gene expression at the second hour and after 24 h. In particular, as Figure 6a shows, an increased expression level of a small number of genes was observed in all groups after 2 h. Among the markers of osteogenic differentiation, ALPL, BGLAP, COL1A1, TNF, and VDR genes had an increased concentration of mRNA. Some other single genes in the groups of chromatin and chromosome modulators (KAT2A and TERT), markers of asymmetric division (WNT1), stem reduction (CD34), antiapoptosis (NOS2 and TRAF2), necrosis (JPH3), and proapotosis (CD40) were characterized by a high level of expression. The expression of the JAG1 and NOTCH1 (NOTCH signaling), COL3A1 and EGFR (osteogenic differentiation), and FOXI1 genes (markers of necrosis) was significantly lower than the control level.

Increased gene expression was observed in a cluster of chromatin and chromosome modulators (KAT2A and TERT), regulators of symmetric cell division (NUMB and PARD6A), osteogenic differentiation (FGFR1, IGF1, IGFR1, and VDR), self-renewal (SOX1), cancer stem cell markers (ALDH1A1 and CD24), stemness loss (PITCH1), migration and metastasis (IL8 and SNAI1) and pluripotency (NANOG and POU5F1). A gene from the necrosis marker group, FOXI1, also had an increased mRNA concentration. Transcription levels were significantly reduced in several genes (HDAC2, NOS2, and MCL 1) from different groups simultaneously.

**Figure 7:**
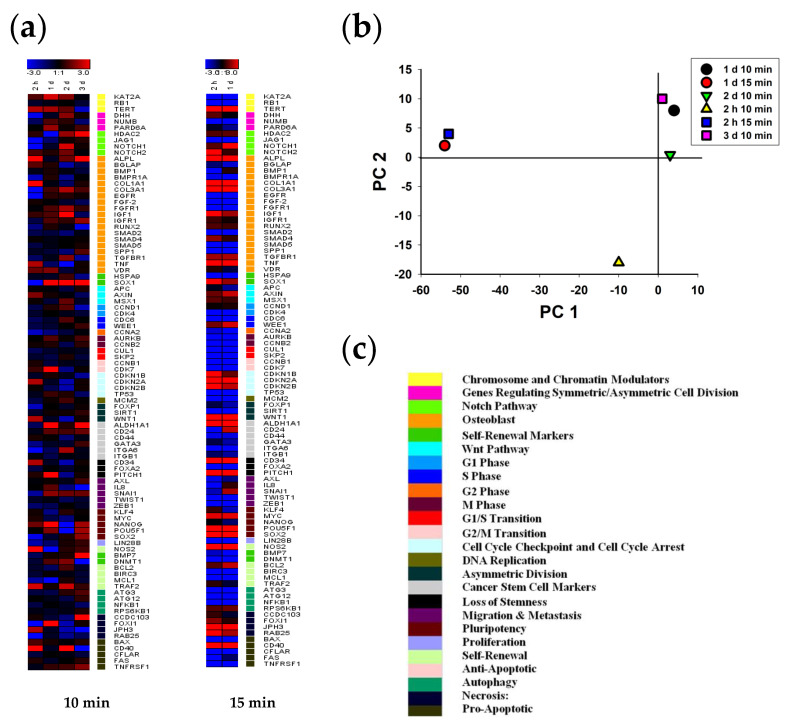
Heat map of gene expression in MNNG/HOS cells treated with 10 and 15 min CAAP irradiation after 2 h, 24 h, 48 h and 72 h of incubation (the deviations are shown in a logarithmic scale with a base of 2): (**a**) the intensity scale of the standardized expression values ranges from −3 (green: low expression) to +3 (red: high expression, with 1:1 intensity value (black) representing the control (non-treated); (**b**) principal component analysis (PCA) of qRT-PCR data for MNNG/HOS cells treated with 10 and 15 min CAAP irradiation after 2 h, 24 h, 48 h and 72 h; (**c**) cluster groups of genes and their functionality. The belonging of genes to different clusters and markers is shown using the color scale which is to the right of the diagrams. A validated database for gene ontology annotations is presented in Table S1.

The gene transcription in the cluster of chromatin and chromosome modulators (KAT2A and TERT), regulators of symmetric cell division (DHH), osteogenic differentiation (ALPL, COL3A1, FGFR1, IGF1, and TGFBR1), and self-renewal (SOX1) continued to increase two days after irradiation. Stimulation of gene expression was also observed in the NOTCH signaling group (HDAC2, NOTCH1, and NOTCH2). The expression level of single genes either increased (MSX1, CCND1, ITGA6, SNAI1, DNMT1, TRAF2, and CD40) or decreased (BGLAP, BMPR1A, COL1A1, SMAD2, TNF, WNT1, CD44, PITCH1, IL8, and TWIST1) in the remaining groups. The decrease in the number of transcripts of genes controlling cell proliferation (CDK7, CNB2, CDKN2A, and CDKN2B), pluripotency markers (MYC, NANOG, and POU5F1), and necrosis markers (CCDC103, FOXI1, JPH3, and RAB25) is noteworthy. PCA analysis is shown in Figure 7b.

On the third day after CAAP irradiation, the cancer cells maintained a high level of transcription for some genes: HDAC2 (NOTCH signaling), ALPL and IGFR1 (osteogenic differentiation markers), SOX1 (self-renewal markers), ALDH1A1 and CD24 (cancer stem cells markers), SNAI1 (migration and metastasis markers), and for pluripotency markers (NANOG, POU5F1, and SOX2), the necrosis marker (CCDC103), and the pro-apoptotic factor TNFRSF1.

An increase in the duration of MNNG/HOS cell irradiation to 15 min was accompanied by a sharp drop in the expression level of most of the studied genes (Figure 6a). Moreover, the transcription of many genes was not even detected. At the same time, very high levels of the expression of some genes were observed, which persisted even after a day of cultivation. In particular, these were chromatin and chromosome modulator genes (TERT), NOTCH signaling (HDAC2, NOTCH1, and NOTCH2), osteogenic differentiation (ALPL, COL1A1, COL3A1, IGF1, SMAD4, TGFBR1, RUNX, and TNF), self-renewal (SOX1), WNT signaling (AXIN and MSX1), cell cycle arrest checkpoints (CDKN1B, CDKN2A, and CDKN2B), and asymmetric division and cancer stem cell markers (WNT1 and ALDH1A1). Furthermore, the genes in the groups of stemness loss markers (CD34 and PITCH1), pluripotency (MYC, POU5F1 and SOX2), anti-apoptosis (NOS2, BCL2), necrosis (FOXI1, JPH3 and RAB25), and pro-apoptotic factor CD40 had very high levels of expression.

We could not trace the further course of changes in the gene expression level in MNNG/HOS cells after 15 min of CAAP irradiation, because a significant fraction of cells died and detached from the surface of the culture plates on the second and third days of cultivation.

Thus, it can be concluded that CAAP irradiation of normal and cancer cells is realized, including through various signaling pathways, expressed in changes in the level of expression of genes responsible for the cellular response to oxidative stress and the development of apoptosis. At the same time, given that the basal levels of expression of the selected genes differ significantly, we can say that the same CAAP exposure time will differ significantly depending on a cell type.

## 3. Discussion

In our study we have shown that it is possible to create a cold argon atmospheric plasma treatment mode which does not only slow down the growth of stem cells, but also accelerates it. In turn, the growth of cancer cells was significantly slowed down with the duration of irradiation of up to 15 min. A longer exposure of 20 min caused a slowdown in growth of both cancer and stem cells.

The inhibitory effects of CAAP on cell growth in vitro have been described in many types of cell cultures, both primary and cancer [23,24,25,26,27]. Cancer cells are less resistant to CAAP then normal ones [28]. The greater tendency to apoptosis after CAP exposure in malignant epithelial cells compared to normal ones has been shown in a study by Kalghatgi et al. [29]. A similar hypersensitivity was detected also in Volotskova’s study, where it was shown that cell cultures of papilloma line 308 and carcinoma PAM212 are more sensitive to the effects of helium plasma than the normal keratinocytes [30]. Tuhvatulin et al. showed that plasma treatment of the HST116-p53-Gal cell line with the MicroPlaSter device leads to the activation of p53-controlled apoptosis, while the same irradiation of the 293 NF κB-Gal cell line leads only to the shutdown of the NF-κB regulation [31].

We found not only greater sensitivity and damage of cancer cells compared to primary cell cultures, but we also established modes that promote the growth inhibition of cancer cells and at the same time stimulate the growth of stem cells.

The vast majority of researchers believe that the active forms of oxygen and nitrogen are a possible active principle of CAP irradiation. Excessive concentration of the reactive oxygen species leads, in particular, to the suppression of cell proliferation [32,33,34,35,36]. It is obvious that the deceleration of cancer cell growth and the cell death at prolonged exposure to plasma irradiation is caused by a ROS generation. Here, we showed the ROS generation in cancer and stem cells after 15 min of irradiation using the HyPer intracellular sensor and the H_2_DCFDA dye. It is well known that cancer cells are more sensitive to the presence of pro-oxidants since they generate high levels of ROS themselves [37,38]. The CAP treatment of these cells leads to the formation of critical ROS levels, which explains their higher sensitivity to the cold plasma effects.

The cell sensitivity to ROS is due to the function of the system of Nrf2, a transcription factor that regulates the expression of antioxidant defense proteins (glutathione (GSH), glutathione reductase (GSR), glutathione S-transferase (GST), etc.) [39]. This antioxidant defense system is also involved in the biological response to the CAP effects. For example, cold plasma irradiation of the wound surface activates Nrf2 and translocates it into the skin cell nucleus, which leads to increased proliferation of kerataninocytes and dermal fibroblasts [40]. It is likely that Nrf2 is involved in the adaptive response to CAP exposure in MSCs. Nrf2 activity is often high in cancer cells because of increased ROS generation [41]. Therefore, Nrf2 cannot provide protection of a cancer cell when the ROS concentration increases additionally due to CAP. It leads to oxidative distress and cell death.

As a result of our study of proliferation and cell death after exposure to CAAP, we found that the irradiation under the same modes suppresses the cell cycle and leads to the death of the cancer MNNG/HOS cell population, while the MSCs were found to be more stable. Thus, after irradiation of cell cultures with helium plasma, the cell cycle arrest at the G1/S point and death of papilloma and carcinoma cells were observed, while the growth and proliferative activity of normal keratinocytes did not differ from the control [42,43,44].

Moreover, we found the cell cycle arrest of cancer cells under the same modes of CAAP irradiation, which not only did not damage the MSCs, but also contributed to the activation of their proliferation. Blocking effects occurred in the G1/S or G2/M checkpoints depending on the irradiation dose and the time after exposure. Moreover, the expression of genes responsible for the cell cycle passage was significantly decreased in cancer cells after exposure to CAAP. On the contrary, the transcription level of the genes ensuring the cell cycle arrest was very high. We showed the expression of genes responsible for proliferation, DNA replication, and the transition between cell cycle phases in MSCs irradiated with CAAP under the discovered proliferation-stimulating mode. It is likely that a stimulation of the expression of genes responsible for cell proliferation is a key factor that provides an increased level of cell culture proliferation and growth. In addition to an increase in the proliferative activity of MSCs after exposure to CAAP, we found increased levels of osteogenic differentiation marker expression. A similar gene induction after osteogenic differentiation was observed in MNNG/HOS osteosarcoma cells after exposure to CAAP (in particular, the ALPL, IGFR1, and COL3A genes induction). The transcription level of these genes increased significantly after 15 min of exposure. This fact may indicate that the proliferation of cancer cells can be stopped by inducing their differentiation.

Thus, we have shown that CAAP treatment is able to activate the processes of cell differentiation not only in stem cells, but also in cancer ones. As reported previously [45,46], the activation of proliferation and differentiation of stem cells by CAP can be due to NO which is also formed in sufficient quantities in the inert gases’ cold plasma.

We determined that MSCs died mainly by apoptosis after exposure to CAAP. The FACS plots showed cells in early and late apoptosis and there were practically no necrotic cells. However, cancer cells died mainly due to necrosis. The initiation of cell death by apoptosis or necrosis after CAP irradiation was reported elsewhere in some types of cancer cells, for example, melanoma and various carcinomas, as well as in normal fibroblasts, muscle cells and keratinocytes [47,48,49]. The nature of the response (necrosis or apoptosis) depended on the exposure dose, intensity, duration, and composition of the CAP gas phase. Sufficiently long-term and high-intensity exposure to CAP leads, as a rule, to necrotic processes, while a less intensive irradiation triggers the apoptosis mechanism [50]. In most cases after moderate CAP irradiation, there was a fragmentation of genomic and mitochondrial DNA and a release of cytochrome C (typical apoptosis signs) [51]. An increase in oxygen concentration in the mixture of gases involved in the formation of CAP, as a rule, enhanced the biological effects of CAP [52].

Thus, CAP irradiation can activate the necrosis and apoptosis processes both by exogenous activation and endogenous activation. Most studies on CAP describe the activation of caspase 3 after 8–16 h of exposure [53]. Using a genetically encoded sensor of caspase 3, we found that this protein is activated by proteolytic cleavage even earlier—1 h after exposure to CAAP. The development of the apoptosis or necrosis activation was found to be associated with an increase in the expression of genes that regulate apoptotic processes. We observed a significant increase in the transcription of these genes in cancer cells after 15 min of CAAP irradiation.

Prolonged MSC and cancer cell exposure to CAAP in our experiments was accompanied by them losing their adhesive properties and detaching from the surface. It is well known that adhesion in eukaryotic cells depends on the well-coordinated functioning of many proteins that play an important role in the processes of cell migration, proliferation and differentiation. In general, adhesion proteins can be divided into calcium-dependent (cadherins) and calcium-independent (integrins and CAM). Changes (increase or decrease) in the expression of adhesion proteins (α2, α5, α6, β1 and β3 integrins, and E-cadherin) in response to CAP exposure are described in the HaCaT keratinocyte cell cultures [54,55,56], and fibroblasts [57].

We studied the expression of some genes responsible for a cell adhesion in MSCs and showed that 10 min of irradiation mainly stimulated the expression of integrins (ITGA6 and ITGB1), while the expression of these genes in cancer cells is inhibited and, after 15 min of irradiation, is nearly undetected. Thus, exposure to CAAP contributed to the loss of cell adhesion properties that are associated with the impaired expression of cell adhesion proteins. Moreover, these effects are more pronounced in MNNG/HOS cells. It is believed that changes in the number of adhesion protein molecules on the cell surface may be due to an increase in ROS production in cells after CAP irradiation [44,54,58]. For example, it has been shown that hydrogen peroxide at a concentration of 100 µM promotes a loss of cell adhesion to the surface [59,60].

Based on the data we have obtained and information from the literature, the CAP effect on MSCs and cancer cells can be described within the framework of the hormesis concept [61,62]. The concentrations of ROS and RNS at low doses of CAP irradiation are non-critical, and the cell response is expressed by the stimulation of vital processes (the first response phase). In the case of MSCs, it leads to the activation of proliferation and differentiation, while in the cancer cells, the effect is absent or there is a growth slowdown. The increasing of the plasma irradiation dose boosts the concentrations of ROS and RNS to critical values. As a result, the second response phase in the form of cell suppression and damage is formed, which leads to the massive death and suppression of cell growth in the case of cancer cells. The sensitivity of different cell types to ROS and RNS which are formed after the CAP irradiation can be used for therapeutic purposes for targeted destruction of cancer cells without affecting normal cells. Nevertheless, it is important to carefully select the optimal conditions and the dose of CAP irradiation which could provide such a therapeutic effect.

## 4. Materials and Methods

### 4.1. Cell Culture

Experiments were made using primary cultures of normal human mesenchymal stem cells and a cancer MNNG/HOS (Human Caucasian osteosarcoma) cell line. Mesenchymal stem cell (MSC) culture was isolated from the abortive material obtained from an institution licensed by the Ministry of Health of the Russian Federation operating within the Russian Federation legislative framework. Aseptically selected tissues were washed with DMEM culture medium, crushed to 0.5 mm × 0.5 mm fragments, and treated with 0.25% trypsin solution for 15–20 min at 37 °C. The cells were collected by 2 min centrifugation at 1500 g and cultivated in a DMEM medium containing 10% fetal bovine serum (FBS). The mixed primary culture was separated based on the difference in the adhesion times of various cell types to the culture surface [63]. Cell cultivation was performed in DMEM/F-12 medium (1:1, Life Technologies, Carlsbad, California, USA) containing 10% FBS, 2 mM of L-glutamine, 100 U/mL of penicillin, 100 µg/mL of streptomycin, and vitamin solution (PanEco, Moscow, Russia). The cells were cultivated at 37 °C in an atmosphere containing 5% CO_2_. As the cells grew and reached a sub-confluent state, they were treated with a 0.25% trypsin-EDTA solution and passed into new culture flacks in a ratio of 1:2. The 5th passage was used for the experiments.

All the used cells were phenotyped by CD44, CD90, CD105 antigens (presence) and CD34, CD45, HLA-DR antigens (absence).

Cancer MNNG/HOS cells were cultivated in DMEM/F-12 medium (1:1, Life technologies, Carlsbad, California, USA) containing 10% FBS, 2 mM of L-glutamine, 100 U/mL of penicillin, 100 µg/mL of streptomycin, and vitamin solution (PanEco, Moscow, Russia). The cells were cultivated at 37 °C in an atmosphere containing 5% CO_2_. As the cells grew and reached a sub-confluent state, they were treated with a 0.25% trypsin-EDTA solution and passed into new culture flacks at a ratio of 1:2.

### 4.2. CAAP Treatment Procedure of Cell Culture In Vitro

To generate low-temperature argon plasma (CAAP), a MicroPlaSter setup (microwave generator of low-temperature gas plasma) was used, which was made by AD’TEK Plasma Technology Co. Ltd. (Hounslow, Middlesex, UK). Access to this setup was provided by Joint Institute for High Temperatures RAS (Moscow, Russia) with the assistance of Professor Gregor Morfill from the Max Planck Institut für extraterrestrische Physik (Ringberg, Germany). The gas plasma flow was formed in a jet of highly purified argon (99.998%) flowing through a microwave burner at a speed of 2.2 L per minute. The temperature of the outgoing gas jet was below 40 °C.

For the CAAP irradiation, cells were seeded in 96-well plates. As they grew and reached a 10–20% confluence (about a day after seeding), the medium was changed. Then, the cells were irradiated with CAAP. The plate was divided into 2 parts for the experiment. The first part was irradiated with a cold plasma, the second one was covered with a light-tight lid and used as a control. All experiments were done at 8 cm distance from the emitter aperture. The exposure dose was varied by changing the irradiation duration (5–20 min). The cells were irradiated once. After irradiation, the cultural medium was changed, and cells were placed in an incubator. Then, we performed a complex analysis of biological effects (confluence analysis, proliferation rate, flow cytometry, apoptosis analysis, ROS formation, and fluorescent microscopy) and extracted mRNA for analyzing the gene expression.

### 4.3. The Analysis of Cell Proliferation Rate in Normal and Cancer Cells

The cell culture growth rate was estimated by the dynamics of confluence changing (occupied area) using a flatbed reader Clone Pix Imager (Molecular Devices, San Jose, CA, USA). The screening was performed every 24 h for 5 days. Each experimental point is accounted for at least 4 measurements (for 96 wells each).

### 4.4. Flow Cytometry (FACS Analysis)

To obtain the suspension, the cells were treated with a 0.25% trypsin-EDTA solution. The suspension with a cell concentration of 10^6^ was washed twice and resuspended in 1 mL of DMEM/F12 medium. Then, 500 µL of the suspension was taken from the sample and centrifuged at 1500 g for 5 min. The supernatant was removed and resuspended in 100 µL of annexin-binding buffer (0.01 M HEPES (pH = 7.4); 0.14 M NaCl; 2.5 mM CaCl_2_) containing 1 µg/mL of Annexin V-FITC (Biotium, Fremont, CA, USA), 1 µg/mL of propidium iodide (PI) (Biotium, Fremont, CA, USA), and 10 µg/mL of Hoechst 33342 (Sigma, St. Louis, MO, USA) (Chan et al., 2012). The cells were incubated in darkness at room temperature for 15–20 min. The suspension volume was adjusted to 1 mL with annexin-binding buffer and placed on ice (the samples were stored in this state until analysis for 1 h). Then, the cell suspension was filtered through a 100 µm filter (Beckman Coulter, Brea, CA, USA) and analyzed by a MoFlo XDP cell sorter (Beckman Coulter, Brea, CA, USA) using 355, 488, and 635 nm lasers. FACS plots were constructed based on the parameters of SSC, FSC, and fluorescence channels FL1 (FITC), FL3 (PI), and FL6 (Hoechst 33342). The obtained data were analyzed using the program Summit 5.0.1.3804 (Dako, Via Real Carpinteria, CA, USA). FACS plots were used to determine the number of cells in different cell cycle phases, the number of cells that have not undergone apoptosis, and the number of cells in early and late apoptosis and necrosis.

A cell culture treated with an apoptosis inducer staurosporin was used as a positive control [64]. For this purpose, staurosporin (Biotium, Fremont, CA, USA) was added to the culture medium 12–24 h before cell staining and analyzing. The final concentration of staurosporin was 100 nM. The cells cultured under standard conditions were used as a negative control. A suspension of an unpainted control cell culture was used to calibrate the flow cytometer and to adjust the compensation.

### 4.5. Reverse Transcription Polymerase Chain Reaction (RT-PCR)

mRNA was pulled using a magnetic particle kit according to the attached protocol (Sileks, Moscow, Russia). Reverse transcription was performed using a Sileks kit (Moscow, Russia) with an oligo-dt primer according to the attached protocol. The resulting cDNA served as a template for real-time PCR. The reaction was performed in a reaction mixture with SybrGreen (Syntol, Moscow, Russia) using a CFX-96 amplifier (BioRad, Irvin, CA, USA) or ABI 7500 Fast Real-Time PCR System (Applied Biosystems, Waltham, MA, USA). The expression of 93 genes responsible for 25 key cellular processes was determined (Table S1). The analyzed genes were selected from the database http://www.qiagen.com (accessed on 12 June 2019) for PCR profiling of various biological processes. The level of gene transcription was normalized according to the average expression levels of the house-keeping genes β-actin, rplp0 (ribosomal protein, large, P0), and gapdh (glyceraldehyde-3-phosphate dehydrogenase). Gene-specific primers were selected in the Primer Express software (Applied Biosystems, Waltham, MA, USA). Each measurement was made 2 times (internal repeat) and averaged over 3 independent samples. The control was a sample without a reverse transcription stage.

The expression data analysis and principal component analysis (PCA) were performed using the online service http://www.qiagen.com (accessed on 25 September 2020) and the Genesis program [65]. We took into account only the results for which changes in the gene expression were observed at *p* < 0.05.

### 4.6. ROS Determination in In Vitro

Reactive oxygen species (ROS) in MSCs were determined by 2,7-dichloro—dihydrofluorescein-diacetate-acetyl ether (H_2_DCFDA) (Biotium, Fremont, CA, USA). Cells were prepared in the manner described above, while in this case 96-well plates designed for cell fluorescence analysis were used for cultivation (Corning, Midland, NC, USA). After the culture reached 40–50% confluence, the culture medium was changed to PBS and 10 µM of H_2_DCFDA was added (Biotium, Fremont, CA, USA). The cells were incubated for 60 min at 37 °C in a humidified atmosphere containing 5% CO_2_ in darkness. Then, the medium was changed to fresh PBS and the cells were exposed to CAAP as described above. The positive control group was obtained after 30 min cell incubation in 100 µM of H_2_O_2_. The level of H_2_DCFDA fluorescence in cells was detected by a fluorescent reader Infinity 200 (Tecan, Grödig, Austria).

### 4.7. Apoptosis and ROS Detection in Cancer MNNG/HOS Cells

A genetically encoded sensor HyPer-cyto is widely used for the lifetime observation of ROS generation in cancer cells [66]. A genetically encoded sensor based on caspase-3 Casper3-GR is widely used to detect apoptosis in cells in vitro [67]. We used both of these genetically encoded sensors for the analysis of ROS formation and apoptosis development in MNNG/HOS cells. Vectors encoding these proteins were purchased from Eurogen (Moscow, Russia).

The MNNG/HOS line cells were seeded in specially prepared 14-well plates cut from a solid 96-well plate (Costar, Midland, NC, USA), or in 8-well cover glasses (Eppendorf, Enfield, CT, USA). Then the cells were incubated to achieve 50–60% confluence. Cell transfection with the vector was performed with Lipofectamine ^®^ 2000 (Thermo Fisher Scientific Inc., Waltham, MA, USA) or TurboFect ™ (Thermo Fisher Scientific Inc., Waltham, MA, USA) according to the attached protocol. The final concentration of the plasmid was 1 ng/µL and the concentration of the transfecting agent was 0.5 µL per 100 µL of the medium. The cells were screened for the fluorescence of the expressed fluorescent protein using an inverted fluorescence microscope AxioVert A1 (Carl Zeiss, Oberkochen, Germany). The in vivo study of the dynamics of intracellular processes in transfected cells carrying genetically encoded sensors of hydrogen peroxide and caspase-3-dependent apoptosis was done using a microscopic complex for cell imaging, Cell Observer A1 (Carl Zeiss, Oberkochen, Germany).

After CAAP irradiation, cells were placed in the chamber incubator of the Cell Observer system (Carl Zeiss, Oberkochen, Germany). Cells carrying the HyPer protein were photographed using a FITC detection filter (Ex/Em = 490/525 nm) for 10 min after CAAP exposure. The cells with 100 µM of H_2_O_2_ were used as a positive control. To visualize the Casper3-GR protein, cells were photographed using a filter for FITC and Rhodamine (Ex/Em = 511/534). Images were taken for 3 h with an interval of 2 frames/min. Staurosporin (100 nM) was used as a positive control for an apoptosis activation. Unaffected cells were used as a control in all cases.

The total fluorescence of individual cells was determined in the obtained images using the ImageJ software (NIH, Bethesda, MD, USA). Both green and red fluorescence channels were used before and after CAAP exposure. Then, the ratio change of the fluorescence intensity in the green and red channels was evaluated. The obtained data were averaged according to the results of measurements of 50 cells per each experimental group.

### 4.8. Statistical Analysis

The obtained data were processed statistically using one-factor variance analysis ANOVA and the student’s parametric criterion. Data processing was performed using Sigma-Plot 9.11 software (Systat Software Inc., Erkrath, Germany). The results were presented as average values ± confidence interval (95%).

## Data Availability

Data sharing not applicable.

## Supplementary Materials

Supplementary materials are available online at https://www.mdpi.com/article/10.3390/ijms22136797/s1.

## Abbreviations

CAP	Cold atmospheric plasma
CAAP	Cold atmospheric argon plasma
MSCs	Mesenchymal stem cells
HF	Human embryonic fibroblast
MNNG/HOS	Human osteosarcoma cancer cell line
H_2_DCFDA	2,7-dichloro-dihydrofluorescein-diacetate-acetyl ether
ROS	Reactive oxygen species
RNS	Reactive nitrogen species
H_2_O_2_	Hydrogen peroxide
FITC	Fluorescein isothiocyanate
Nrf2	Nuclear factor erythroid 2–related factor 2
NO	Nitric oxide
