# Supplementary material for: Dose-Dependent Effects of Cold Atmospheric Argon Plasma on the Mesenchymal Stem and Osteosarcoma Cells In Vitro
Source: Int J Mol Sci. 2021 Jun 24;22(13):6797. doi: 10.3390/ijms22136797 (PMC8269077; doi:10.3390/ijms22136797)
Supplement: Supplementary file 1 [file ijms-22-06797-s001.zip › ijms-1249701-supplementary.pdf]

Supplementary Materials

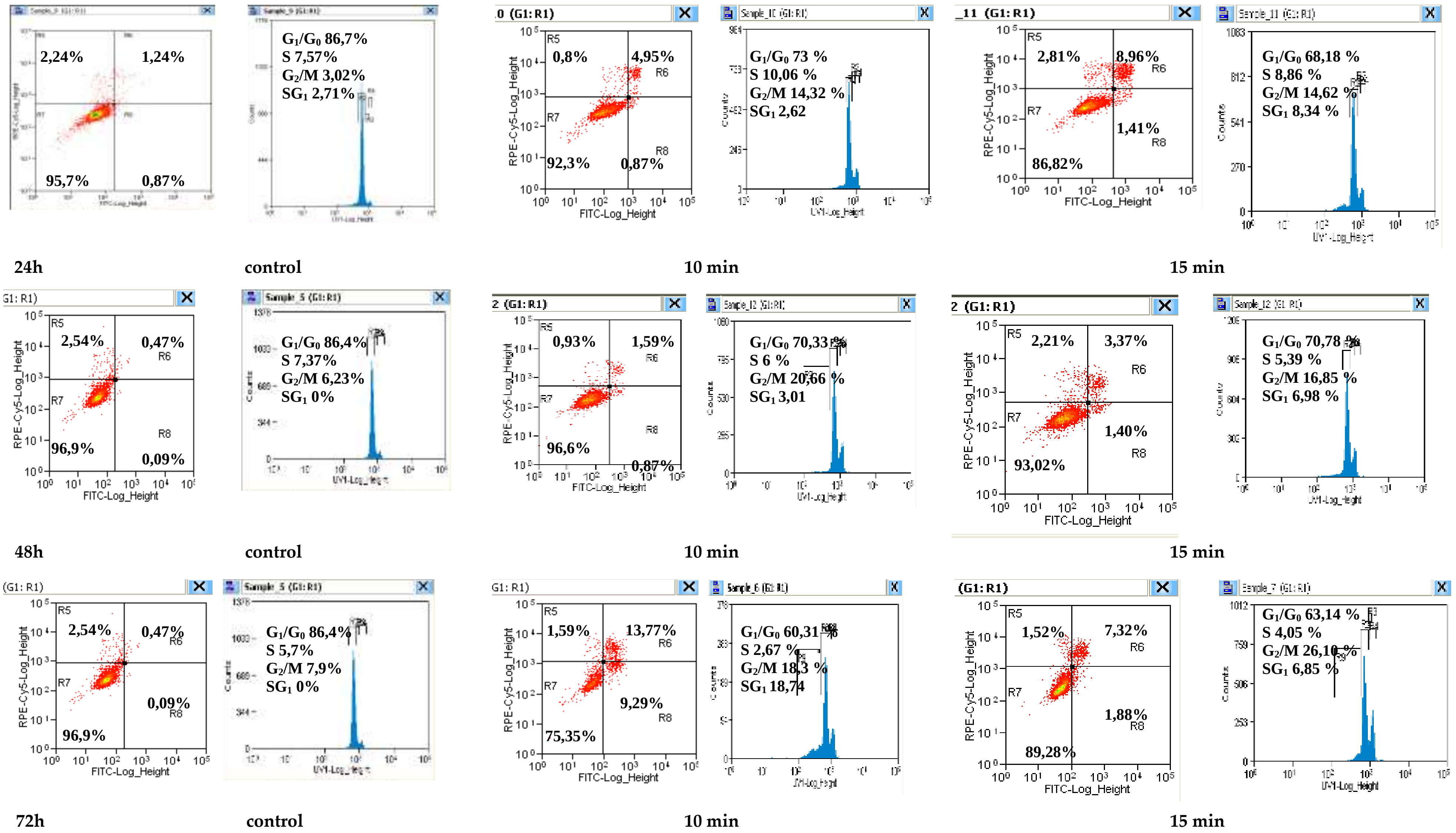

a

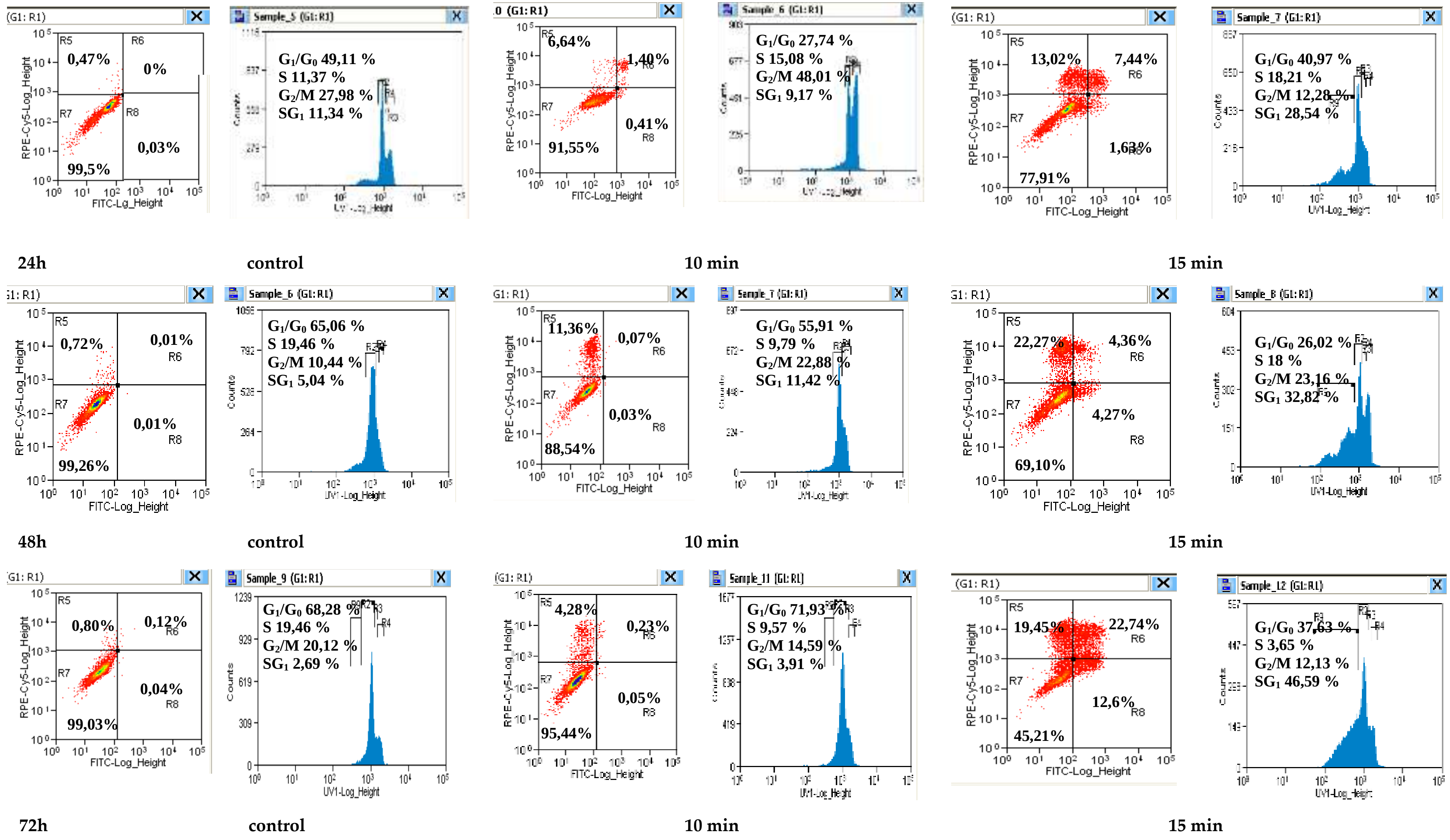

b

Figure. S1. The flow cytofluorometry of cell populations after CAAP irradiation after 24, 48 and 71 h with the 10 and 15 min CAAP exposure time: (a) MSCs (HF); (b) MNNG/HOS. Red dots on the left side of each diagram show the distribution of cells by quadrant depending on their state: R5 – necrosis, R6 – late apoptosis, R7 – living cells, R8 – early apoptosis. The histograms on the right side show cell distribution by cell cycle phases: G_1/G_0, S, G_2/M, and SG_1 (G_1 subphase). The digital data on the histograms are the average values for 3 independent experiments. On the left, the histograms of cell distribution by cell cycle phases are shown in blue; G1/G0, S, G2/M, and SG 1 (G1 subphase). The digital data on the histograms are the average values of 3 independent experiments.

Table S1. Selected gene groups for RT-PCR analysis.

Gene function	Abbreviation	Gene name	NCBI Reference Sequence	Primer F	Primer R
Chromosome and Chromatin Modulators	KAT2A	K(lysine) acetyltransferase 2A	NM_021078.2	CATCCGCTTCCCCATTGAC	AGGTCGGCCACAAAGAGCTT
Chromosome and Chromatin Modulators	RB1	retinoblastoma 1	NM_000321.2	CACCTCCCATGTTGCTCAAAG	ATCCGTGCACTCCTGTTCTGA
Chromosome and Chromatin Modulators	TERT	telomerase reverse transcriptase	NM_198253.2	CTCACCCACGCGAAAACCT	CCACTGTCTTCCGCAAGTTCA
Genes Regulating Symmetric/Asymmetric Cell Division	DHH	desert hedgehog	NM_021044.2	TTTGGCCATTGCCGTGAT	CGTCCCAGCCCTCAGTCA
Genes Regulating Symmetric/Asymmetric Cell Division	NUMB	numb homolog (Drosophila)	NM_003744.5	CCAGGCCGGTCATAGACGTA	GCCCGGACGCTCTTAGACA
Genes Regulating Symmetric/Asymmetric Cell Division	PARD6A	par-6 family cell polarity regulator alpha	NM_016948.2	CCGGCCACCCTTGCTAA	CCACGTCTATGACTGAGGAAACCT
Notch Pathway	HDAC2	histone deacetylase 2	NM_001527.3	GGAGAAGATTGTCCAGTGTTTGATG	GTTTAACTTCACAGCTCCAGCAACT
Notch Pathway	JAG1	jagged 1	NM_000214.2	TTCCCTTGCTGAGCTCTGTCTT	TCCGCAGGCACCAGTAGAAG
Notch Pathway	NOTCH1	notch 1	NM_017617.3	GCGGGTCCACCAGTTTGA	CCGCAGAGGGTTGTATTGGTT
Notch Pathway	NOTCH2	notch 2	NM_024408.3	CCACCTGAAGGGAAGCACATAA	GATGAGCTGGAAAGTCACAATGG
Osteoblast	ALPL	alkaline phosphatase, liver/bone/kidney	NM_000478.4	CATGCTGAGTGACACAGACAAGAA	ACCGCCCACCACCTTGTAG
Osteoblast	BGLAP	osteocalcin (bone gamma-carboxyglutamate (gla) protein)	NM_199173.4	GGTGCAGAGTCCAGCAAAGG	GCGCCTGGGTCTCTTCACTA
Osteoblast	BMP1	bone morphogenetic protein 1	NM_006129.4	AGGGCAATGATGTGTGCAAGT	GCCATGCAGCTTGGAGTCA
Osteoblast	BMPR1A	bone morphogenetic protein receptor, type IA	NM_004329.2	TCAGCGAACTATTGCCAAACAG	TTGCCCATCCATACTTCTCCAT
Osteoblast	COL1A1	collagen, type I, alpha 1	NM_000088.3	CGATGGCTGCACGAGTCA	CAGGCGGGAGGTCTTGGT
Osteoblast	COL3A1	collagen, type III, alpha 1	NM_000090.3	TGGAGGATGGTTGCACGAA	GTAGTCTCACAGCCTTGCGTGTT
Osteoblast	EGFR	epidermal growth factor receptor	NM_005228.3	CTGAGCTCTCTGAGTGCAACCA	CAGCTTTGCAGCCCATTTCT

Osteoblast	FGF-2	fibroblast growth factor 2 (basic)	NM_002006.4	CGGTCAAGGAAATACACCAGTTG	GCCCAGGTCCTGTTTTGGAT
Osteoblast	FGFR1	fibroblast growth factor receptor 1	NM_015850.3	TGCACCAACGAGCTGTACATG	CTGCTTGAAGGTGGGTCTCTGT
Osteoblast	IGF1	insulin-like growth factor 1 (somatomedin C)	NM_000618.3	TGCTTCCGGAGCTGTGATCT	GCTGACTTGGCAGGCTTGAG
Osteoblast	IGFR1	insulin-like growth factor 1 receptor	NM_000875.3	ACGGCATGGCATACCTCAAC	TTCGGCTACCATGCAATTCC
Osteoblast	RUNX2	runt-related transcription factor 2	NM_004348.3	GTATGTCCGCCACCACTCACTAC	GAAGGGTCCACTCTGGCTTTG
Osteoblast	SMAD2	SMAD family member 2	NM_005901.5	GACACCAGTTTTGCCTCCAGTATT	TCCAGAGGCGGAAGTTCTGTT
Osteoblast	SMAD4	SMAD family member 4	NM_005359.5	CATTGGATGGGAGGCTTCAG	TCCAGAGACGGGCATAGATCA
Osteoblast	SMAD5	SMAD family member 5	NM_005903.6	AGGAACCTGAGCCACAATGAAC	GTTGTTGGGCTGGTGGAAAG
Osteoblast	SPP1	osteopontin-1 (secreted phosphoprotein 1)	NM_000582.2	TTCGCAGACCTGACATCCAGTA	CCATTCAACTCCTCGCTTTCC
Osteoblast	TGFBR1	transforming growth factor, beta receptor 1	NM_004612.2	TGTGCTTCGTCTGCATCTCACT	TGGCACTCGATGGTGAATGA
Osteoblast	TNF	tumor necrosis factor	NM_000594.3	AGGCCAAGCCCTGGTATGA	GCTGAGTCGGTCACCCTTCTC
Osteoblast	VDR	vitamin D (1,25- dihydroxyvitamin D3) receptor	NM_000376.2	GCTGGACGCCCACCATAA	CTCCACCATCATTCACACGAAC T
Self-Renewal Markers	HSPA9	heat shock 70kDa protein 9 (mortalin)	NM_004134.6	GCCAGAACCACCCCTTCAG	GCTTGGCCGGCATTCC
Self-Renewal Markers	SOX1	Homo sapiens SRY (sex determining region Y)-box 1	NM_005986.2	GGTCAAACGGCCCATGAAC	TGATCTCCGAGTTGTGCATCTT
Wnt Pathway	APC	adenomatous polyposis coli	NM_000038.5	AGCAGCTCAAGCAAACACAGTTC	TAGGGCTTGGGTTGTAATTAAA AGG
Wnt Pathway	AXIN	axin 1	NM_003502.3	AAGAAATGCCAAGAAGGCTGAG T	TCTGGTTCTTCTCCGCATCCT
Wnt Pathway	MSX1	msh homeobox 1	NM_002448.3	CAAGTTCCGCCAGAAGCAGTA	TCTTCACCTGCGTCTCAGTGA
G1 Phase	CCND1	cyclin D1	NM_053056.2	GCATGTTCGTGGCCTCTAAGA	CGGTGTAGATGCACAGCTTCTC
G1 Phase	CDK4	cyclin-dependent kinase 4	NM_000075.3	GCCAGCCGAAACGATCAA	TGCAATTGGCATGAAGGAAA
S Phase	CDC6	cell division cycle 6	NM_001254.3	CCGTAACCTGTTCTCCTCGTGTA	TGTCATCGCCCAGACGTTT
S Phase	WEE1	WEE1 G2 checkpoint kinase	NM_003390.3	GAGGCTGGATGGATGCATTT	AAGCGTTCTGCTCATCAACAGA
G2 Phase	CCNA2	cyclin A2	NM_001237.3	CGGCGCTGCTAGCATTG	CTGCCTTTTCCGGGTTGATA
M Phase	AURKB	aurora kinase B	NM_004217.3	AGCGAGTCCTCCGGAAAGA	GACATTGGAGCGGCTCATG
M Phase	CCNB2	cyclin B2	NM_004701.3	CGACCCTTGCCACTACACTTCT	CTTGGCTAAAGTGTGCTGTTCA AC
G1/S Transition	CUL1	cullin 1	NM_003592.2	ACAACAACGCGGTTACCAAGA	AGTCACAGTATCGAGCCAGCA A
G1/S Transition	SKP2	S-phase kinase-associated protein 2,E3 ubiquitin protein ligase	NM_005983.3	ACAGCACATGGACCTATCGAACT	TGCAACTTGGAACACTGAGAC AGT
G2/M Transition	CCNB1	cyclin B1	NM_031966.3	AACATGGCAGGCGCAAAG	CAATGTCCCCAAGAGCTGTTCT

G2/M Transition	CDK7	cyclin-dependent kinase 7	NM_001799.3	CACACAGGCACTGAAAATGAAG TATT	TGGTCTTGGCAGCTGACATC
Cell Cycle Checkpoint and Cell Cycle Arrest	CDKN1B	cyclin-dependent kinase inhibitor 1B (p27, Kip1)	NM_004064.4	GCCAGCGCAAGTGGAATTT	CTCCACCTCTTGCCACTCGTA
Cell Cycle Checkpoint and Cell Cycle Arrest	CDKN2A	cyclin-dependent kinase inhibitor 2A	NM_000077.4	CCAACGCACCGAATAGTTACG	GGGCGCTGCCCATCA
Cell Cycle Checkpoint and Cell Cycle Arrest	CDKN2B	cyclin-dependent kinase inhibitor 2B (p15, inhibits CDK4)	NM_004936.3	GCGACGACAGATGCTAAAATCC	GAGCAAAGGCCAGCATCCT
Cell Cycle Checkpoint and Cell Cycle Arrest	TP53	tumor protein p53	NM_000546.5	CTGTCCCCGGACGATATTGA	TGGCATTCTGGGAGCTTCAT
DNA Replication	MCM2	minichromosome maintenance complex component 2	NM_004526.3	GCGGGACTATGTGATCGAAGAC	CGCATGACGCTGAACTTCTG
Asymmetric Division	FOXP1	forkhead box P1	NM_001244808.1	AACCCACATGCCTCTACCAATG	GGTGCTCCTCATGGGACAAA
Asymmetric Division	SIRT1	sirtuin 1	NM_012238.4	CGGGAATCCAAAGGATAATTCAG	CCTCGTACAGCTTCACAGTCAA CTT
Asymmetric Division	WNT1	wingless-type MMTV integration site family, member 1	NM_005430.3	TGCGCTTCCTCATGAACCTT	TGGCGCATCTCGGAGAATAC
Cancer Stem Cell Markers	ALDH1A 1	aldehyde dehydrogenase 1 family, member A1	NM_000689.4	GCAGTGAAGGCCGCAAGA	CCCTCTCGGAAGCATCCATAG
Cancer Stem Cell Markers	CD24	CD24 molecule	NM_013230.2	TCCAACTAATGCCACCACCAA	GACCACGAAGAGACTGGCTGTT
Cancer Stem Cell Markers	CD44	CD44 molecule (Indian blood group)	NM_000610.3	CCAAATTCCAGAATGGCTGATC	TGCAATGCAAACTGCAAGAAT C
Cancer Stem Cell Markers	GATA3	GATA binding protein 3	NM_001002295.1	GGACGAGAAAGAGTGCCTCAAG T	GTGGGACGACTCCAGCTTCA
Cancer Stem Cell Markers	ITGA6	integrin, alpha 6	NM_001079818.1	CCTCCCTGAGCACATATTCGAT	TGCCACCCATCCTTGTTGA
Cancer Stem Cell Markers	ITGB1	integrin, beta 1 (fibronectin receptor, beta polypeptide, antigen CD29 includes MDF2, MSK12)	NM_002211.3	CACAGATGCCGGGTTTCACT	AGGTGACATTGTCCATCATTTG G
Loss of Stemness	CD34	CD34 molecule	NM_001025109.1	CTTGGGCATCACTGGCTATTTC	TCTTCGCCCAGCCTTTCTC
Loss of Stemness	FOXA2	forkhead box A2	NM_021784.4	CCACCTGAAGCCGGAACA	TGCTCCGAGGACATGAGGTT
Loss of Stemness	PITCH1	patched 1	NM_001083603.1	TGGTTGTGGGCCTCCTCATA	TCCACGTTGGTCTCGAGGTT
Migration & Metastasis	AXL	AXL receptor tyrosine kinase	NM_021913.4	ACCCAGCCAGACCCTAAGGA	TGTGGAAGGGCAGAGGACAT

Migration & Metastasis	IL8	interleukin 8	NM_000584.3	AGCTGGCCGTGGCTCTCT	CTTGGCAAAACTGCACCTTCA
Migration & Metastasis	SNAI1	snail family zinc finger 1	NM_005985.3	CTTCGCTGACCGCTCCAA	ACACGCCTGGCACTGGTACT
Migration & Metastasis	TWIST1	twist family bHLH transcription factor 1	NM_000474.3	GCGCTGCGGAAGATCATC	TCGCTCTGGAGGACCTGGTA
Migration & Metastasis	ZEB1	zinc finger E-box binding homeobox 1	NM_001128128.2	CACTGGTGGTGGCCCATTAC	TATGGGAGACACCAAACCAAC TG
Pluripotency	KLF4	Kruppel-like factor 4 (gut)	NM_004235.4	TCGCCCGCTCAGATGAAC	CGCATTTTTGGCACTGGAA
Pluripotency	MYC	v-myc avian myelocytomatosis viral oncogene homolog	NM_002467.4	AGTCCTGAGACAGATCAGCAAC AA	GTGTGTTCGCCTCTTGACATTCT
Pluripotency	NANOG	Nanog homeobox	NM_024865.2	ACCAGTCCCAAAGGCAAACA	CTTGACCGGGACCTTGTCTTC
Pluripotency	POU5F1	POU class 5 homeobox 1	NM_002701.5	ACTGCAGCAGATCAGCCACAT	CCACACTCGGACCACATCCT
Pluripotency	SOX2	SRY (sex determining region Y)-box 2	NM_003106.3	CTGCGAGCGCTGCACAT	CCTTCTTCATGAGCGTCTTGGT
Proliferation	LIN28B	lin-28 homolog B	NM_001004317.3	CCGGCAGAGGAGGAATCC	TCCCATGCGCACATTGAA
Anti-Apoptotic	NOS2	nitric oxide synthase 2, inducible	NM_000625.4	GGTGGAAGCGGTAACAAAGGA	TGCTTGGTGGCGAAGATGA
Self-Renewal	BMP7	bone morphogenetic protein 7	NM_001719.2	CAACCTCGTGGAACATGACAAG	TGGAAAGATCAAACCGGAACT C
Self-Renewal	DNMT1	DNA (cytosine-5-)-methyltransferase 1	NM_001130823.1	GCCACTGCACGTGTTTGCT	TCAACCTGGTTATGTTGCTCAC A
Anti-Apoptotic	BCL2	B-cell CLL/lymphoma 2	NM_000633.2	CTGGGATGCCTTTGTGGAACT	AGACAGCCAGGAGAAATCAAA CAG
Anti-Apoptotic	BIRC3	aculoviral IAP repeat containing 3	NM_001165.4	GGACAGGAGTTCATCCGTCAAG	TCTCCTGGGCTGTCTGATGTG
Anti-Apoptotic	MCL1	myeloid cell leukemia 1	NM_021960.4	CACGAGACGGCCTTCCAA	CACTCGAGACAACGATTTCACA TC
Anti-Apoptotic	TRAF2	TNF receptor-associated factor 2	NM_021138.3	GGCCGTCTGTCCCAGTGAT	TTCGTGGCAGCTCTCGTATTC
Autophagy	ATG3	autophagy related 3	NM_022488.4	CCATTGAAAATCACCCTCATCTG	CACCTCAGCATGCCTGCAT
Autophagy	ATG12	autophagy related 12	NM_004707.3	CCCGGGAACAGAGGAACCT	GGAGTGTCTCCCACAGCCTTT
Autophagy	NFKB1	nuclear factor of kappa light polypeptide gene enhancer in B-cells 1	NM_003998.3	GGCTACACCGAAGCAATTGAAG	CAGCGAGTGGGCCTGAGA
Autophagy	RPS6KB1	ribosomal protein S6 kinase, 70kDa, polypeptide 1	NM_003161.3	TGGCATAGAGCAGATGGATGTG	AGAGTTCGGCTGTCGTATTGGA
Necrosis:	CCDC10 3	coiled-coil domain containing 103	NM_213607.2	GCTGCAAGGGCTTGTTTCAG	GCCCCTCCTTCACGGATCT
Necrosis:	FOXI1	forkhead box I1	NM_012188.4	CGCCTCACTCTCAGCCAGAT	CCGGCCTTGCTCTTGTTGTA
Necrosis:	JPH3	junctophilin 3	NM_020655.3	CCAGGATCACTGCCAAAGAGTT	CGCTTCGGCCTCTGGTACT

Necrosis:	RAB25	RAB25, member RAS oncogene family	NM_020387.2	TGTCTTCAAGGTGGTGCTGATC	CGCGTGAATCGGGAGAGTAG
Pro-Apoptotic	BAX	BCL2-associated X protein	NM_004324.3	GTGGCAGCTGACATGTTTTCTG	GCAAAGTAGAAAAGGGCGACAA
Pro-Apoptotic	CD40	CD40 molecule, TNF receptor superfamily member 5	NM_001250.4	ACACTGCCACCAGCACAAATACT	CTGTTTCTGAGGTGCCCTTCTG
Pro-Apoptotic	CFLAR	CASP8 and FADD-like apoptosis regulator	NM_003879.5	GTGTGTATGGTGTGGATCAGACTCA	GGCATGAATCTCCCATGAACA
Pro-Apoptotic	FAS	Fas cell surface death receptor	NM_000043.4	GAATCATCAAGGAATGCACACTCA	AAAGCCACCCCAAGTTAGATCTG
Pro-Apoptotic	TNFRSF1	umor necrosis factor receptor superfamily, member 10a	NM_003844.3	CTGGCGCTTGGGTCTCCTA	TGCGTTGCTCAGAATCTCGTT
Housekeeping	GAPDH	glyceraldehyde-3-phosphate dehydrogenase	NM_002046.5	GTGGAAGGACTCATGACCACAGT	GCCATCACGCCACAGTTTC
Housekeeping	RPLP0	ribosomal protein, large, P0	NM_001002.3	ATGCAGCAGATCCGCATGT	TTGCGCATCATGGTGTTCTT
Housekeeping	Actin	beta-actin	XM_006715764.1	TCGTGCGTGACATTAAGGAGAA	AGCAGCCGTGGCCATCT
